# Commentary : The Developmental Trajectory of the Operational Momentum Effect

**DOI:** 10.3389/fpsyg.2018.02259

**Published:** 2018-11-21

**Authors:** Martin H. Fischer, Alex Miklashevsky, Samuel Shaki

**Affiliations:** ^1^Psychology Department, University of Potsdam, Potsdam, Germany; ^2^The Department of Behavioral Sciences, Ariel University, Ariel, Israel

**Keywords:** embodied cognition, operational momentum, SNARC effect, mental arithmetic, numerical cognition

Recently, Pinheiro-Chagas et al. (2018) studied the development of the operational momentum (OM) which denotes a tendency to accept larger than correct outcomes in addition and smaller than correct outcomes in subtraction. The authors reviewed some theories of OM and derived two competing predictions. First, they described the attentional account, according to which OM results from an overshoot of an attentional spotlight when moving along the spatially oriented mental number line (MNL) in accordance with the magnitude of the second operand. Given that “formal schooling …might consolidate a systematic movement direction during the acquisition of arithmetical skills” (p.3), older children should show more OM. Secondly, they described the compression account of OM according to which linear operations (addition, subtraction) are performed on logarithmically compressed operand representations. Referring to a log-to-linear developmental shift in the placement of numbers on visually presented number lines, they predicted that older children should show less (un-) compression and thus less OM. Their results from 8 to 12-year olds showed a gradual increase of OM starting at 9 years and thus supported the attentional account.

The clear performance pattern reported by Pinheiro-Chagas and colleagues makes a useful contribution to the literature on OM development but their report also misrepresents the state of knowledge about OM. It might leave readers unnecessarily misinformed about the multi-faceted origin of this bias generally, and more specifically about the status of reverse OM for our understanding of cognitive biases in formal reasoning. We draw attention to these points below.

First, the authors acknowledged early OM in 9-month-olds (McCrink and Wynn, 2009) as well as reverse OM in 6-year-olds (Knops et al., 2013), thus recognizing a potential problem with their conclusion of late-emerging and gradually increasing OM. While the authors mentioned the work of Pinhas and colleagues they did not convey its full impact with regard to this point. First, Pinhas and Fischer (2008; see also Shaki et al., 2018) observed larger OM in zero problems (such as 4+0) compared to non-zero problems (e.g., 3+1). This alone could suffice to discredit the compression account because the logarithm of zero is not defined. Thus, the compression account was arguably a mere strawman pitted against the attentional account, although other methodological differences, such as the number format, remain. But if attention shift magnitude is “… a distance corresponding to the magnitude of the second operand” (p. 2), how does this account explain larger OM with zero problems?

Further inconsistencies are reflected in the methods: From an attentional perspective, repeated downward movements of both addends, as well as upward movements of the subtrahend, constitute inconsistencies with the vertical MNL that maps small quantities below larger quantities. Experience with vertical mappings will change over age and might increase the performance consequences of such inconsistencies. More generally, why were operations along a horizontal MNL primed with vertical movements? The fact that subtrahends moved away from the area of interest in the display center removed attention from the place of mentally simulating the outcome, thus impeding subtraction.

Secondly, Pinhas and Fischer (2008) proposed multiple sources of OM, including the operands, the operator, and the result. Taking into consideration evidence from biased quantitative reasoning, estimation heuristics and spatial-numerical associations, we have since developed this proposal into a comprehensive model of arithmetic heuristics and biases (AHAB; see also Shaki and Fischer, 2017; Fischer and Shaki, 2018; Shaki et al., 2018). This model can explain the complete range of findings reported in the literature, including reverse OM, as a weighted contribution from an anchoring effect, an estimation heuristic, and spatial associations of operands and operators. Pinheiro-Chagas et al.'s report created the false impression that reverse OM is an anomaly. Instead it was found repeatedly (Charras et al., 2012, 2014; Knops et al., 2013; Pinhas et al., 2015; Blini et al., 2018) and can be understood as reflecting anchoring bias in non-zero problems. However, anchoring bias increases from fourth to eight grade (Smith, 1999) and this should gradually reduce OM unless other factors compensate for this bias.

One further strength of AHAB is its ability to account for both spatial and non-spatial biases in mental arithmetic, regardless of whether computational uncertainty originated from encoding non-symbolic operators or results, as in studies by Knops and colleagues, or from mapping of perfectly identifiable operators and results onto a continuous response dimension, such as horizontal lines or time intervals (Shaki et al., 2015). It would be interesting to learn whether Pinheiro-Chagas and colleagues replicated the spatial bias in response selection previously observed in this paradigm by Knops et al. (2009).

Finally, the authors mention also an heuristic account of OM: a tendency to accept more than the correct outcome for additions and less than the correct outcome for subtractions because addition leads to “more” and subtraction to “less” (McCrink and Wynn, 2009). They compare it to the attentional account and state that “… the two accounts provide equivalent predictions” (p. 3). This is in conflict with the recent analysis offered in McCrink and Hubbard (2018, p. 240) that “… the use of heuristics is generally increased when attention is decreased”. We think that heuristics are triggered by operators. Yet, OM only emerges late, i.e., when both operator and second operand have been processed (Liu et al., 2017; Masson et al., 2017; Blini et al., 2018). Results obtained from procedures where operators even preceded the first operand (cf. Knops et al., 2009) or multiple quantities are presented during responding (cf. Pinheiro-Chagas et al., 2018) must be interpreted cautiously because the normal ingredients of OM are dis-ordered or diluted.

## Author contributions

All authors listed have made a substantial, direct and intellectual contribution to the work, and approved it for publication.

### Conflict of interest statement

The authors declare that the research was conducted in the absence of any commercial or financial relationships that could be construed as a potential conflict of interest.
